# Asymmetric Synthesis of the Carbon-14-Labeled Selective Glucocorticoid Receptor Modulator using Cinchona Alkaloid Catalyzed Addition of 6-Bromoindole to Ethyl Trifluoropyruvate 

**DOI:** 10.3390/molecules17066507

**Published:** 2012-05-30

**Authors:** Takaaki Sumiyoshi, Daisuke Urabe, Kengo Tojo, Masato Sakamoto, Kazumi Niidome, Norie Tsuboya, Tomoko Nakajima, Masanori Tobe

**Affiliations:** Drug Research Division, Dainippon Sumitomo Pharma Co., Ltd., 33-94 Enoki, Suita, Osaka 564-0053, Japan

**Keywords:** organocatalyst, cinchona alkaloid, asymmetric synthesis, radioisotope labeled compound

## Abstract

We describe in this study the asymmetric synthesis of radioisotope (RI)-labeled selective glucocorticoid receptor modulator. This synthesis is based on optimization of the cinchona alkaloid catalyzed addition of 6-bromoindole to ethyl trifluoropyruvate and Negishi coupling of zinc cyanide to the 6-bromoindole moiety. [^14^C] Labeled (−)-{4-[(1-{2-[6-cyano-1-(cyclohexylmethyl)-1*H*-indol-3-yl]-3,3,3-trifluoro-2-hydroxypropyl}piperidin-4-yl)oxy]-3-methoxyphenyl}acetic acid (−)-**1** was synthesized successfully with high enantioselectivity (>99% *ee*) and sufficient radiochemical purity.

## 1. Introduction

Catalytic asymmetric synthesis, which is one of the important methodologies in modern synthetic chemistry for the preparation of enantiomerically enriched compounds, requires the use of metal catalysts or organocatalysts [1]. Organocatalytic asymmetric reactions, which have recently been developed into a practical synthetic paradigm, have several significant advantages over metal-catalyzed reactions due to the following reasons: (1) minimal sensitivity to moisture and oxygen; (2) low cost and low toxicity; and (3) recyclable and reusable catalysts [2,3,4]. Since the first report of cinchona alkaloids-catalyzed asymmetric reactions by Bredig and Fiske [5], cinchona alkaloids and their derivatives have been recognized as one of the most important organocatalysts in catalytic asymmetric reactions [6,7,8]. Recently, several applications of organocatalysts, including cinchona alkaloids, to Friedel-Crafts reaction have attracted chemists’ attention as useful methods for carbon-carbon formation [9,10,11,12,13,14,15,16,17,18,19,20,21]. Encouraged by these findings, we have reported the use of cinchona alkaloids in the asymmetric addition of 6-cyanoindole to ethyl trifluoropyruvate and applied the reaction to the asymmetric synthesis of glucocorticoid receptor modulator (GRM) (−)-**1** [22].

RI-labeled compounds are widely used in Drug Metabolism and Pharmacokinetics/Absorption, Distribution, Metabolism and Excretion (DMPK)/ADME studies. They help understand drug metabolism in different species, perform DMPK/ADME studies, such as Quantitative Whole Body Autoradiography (QWBA), and evaluate reactive metabolite formation by protein covalent binding tests [23]. Advances in radiochemistry with the expansion of the use of organocatalysts and understanding of the importance of DMPK/ADME studies have led us to synthesize RI-labeled drug candidates using asymmetric reactions with organocatalysts. In support of our drug discovery program for GRM, we required [^14^C]-labeled (−)-**1** with sufficient radiochemical purity. Here, we describe the successful asymmetric synthesis of [^14^C]-labeled (−)-**1**.

## 2. Results and Discussion

**Scheme 1:**
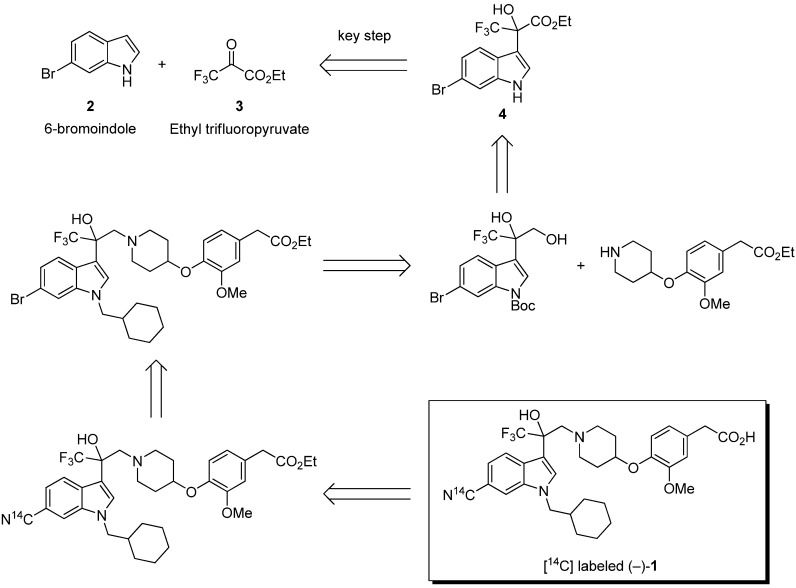
Retrosynthesis of [^14^C] labeled (−)-**1**.

Our approach to the radiosynthesis of (−)-**1** is shown in Scheme 1. The cyano moiety of the indole structure was selected to introduce the most commonly used [^14^C] as radioisotope, because of its chemical and metabolic stability and the availability of [^14^C] cyanide. 

In addition, introduction of the [^14^C] cyanide was optimized as a later step to avoid unnecessary waste of radioactive product. The coupling reaction of cyanide to haloindoles enables this synthetic hypothesis [24]. Based on the findings of our previous report, we used the cinchona alkaloid-catalyzed asymmetric addition of 6-bromoindole to ethyl trifluoropyruvate as a key step. In order to establish a feasible radiosynthetic route, we examined optimization of the cinchona alkaloid catalyzed addition and coupling reaction of cyanide to the 6-bromoindole moiety.

First, we considered preparing the optically pure key intermediate **4** by cinchona alkaloid-catalyzed addition of **2** to **3**. Based on our previous report and Török’s report [25], we focused on the influence of solvents and the bromo group at the C(6)-position of the indole ring (Table 1). Reaction of **2** with **3** in toluene afforded the product (+)-**4** with 83% *ee* in 90% yield (entry 4), which was comparable to that of 6-cyanoindole. Although the reaction proceeded well in diethyl ether (entry 1) and in ethyl acetate (entry 3), enantioselectivity decreased when acetonitrile was used (entry 2). These solvent effects on both yield and enantioselectivity were similar to those obtained with the 6-cyanoindole substrate. 

**Table 1:** Effects of solvents on cinchona alkaloids catalyzed asymmetric addition of **2** to **3** ^[a]^. 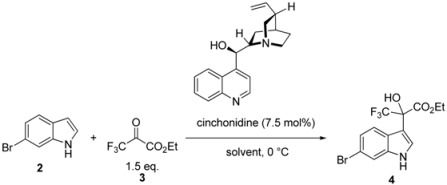

Entry	Solvent	Time (h)	Conversion ^[b]^		Yield (%)	*ee* (%)
			1 h	24 h		
1	Et_2_O	24	83	99	90	89
2	MeCN	24	72	97	87	65
3	EtOAc	24	73	99	89	79
4	Toluene	1	99	−	90	83

^[a]^ *Reaction conditions*: **2** (1.5 mmol), **3** (2.25 mmol) and cinchonidine (37.5 μmol) in solvent (9 mL) at 0 °C; ^[b]^ Conversion was determined on the basis of area % by HPLC (254 nm).

Based on these finding, we selected toluene as solvent, just as we did with 6-cyanoindole, and conducted further optimization of the reaction by examining the effect of temperature and the amount of catalyst (Table 2). Although the yield was maintained with increased reaction temperature, enantioselectivity decreased (entries 1–4). Unlike in the case of 6-cyanoindole, carrying out the reaction even at a very low temperature (−78 °C) afforded the product in high yield (89%), suggesting higher reactivity at the C(3)-position of the 6-bromoindole ring caused by a weaker electron-withdrawing effect of the bromo group than that of the cyano group. On the other hand, the use of 2.5 mol% or 15 mol% of catalyst gave comparable yields and enantioselectivity to the use of 7.5 mol% of catalyst (entries 7 and 8). When the amount of catalyst was reduced to 1 mol%, the yield was maintained, but the enantioselectivity decreased significantly to 71% (entry 6). Interestingly, the initial reaction rate in toluene without the catalyst was comparable to those with catalysts (entry 5) and seems even higher than that of the reaction catalyzed by 1 mol% CD. This result is different from that obtained by Török’s group, in which it was indicated that application of CD as catalyst in Et_2_O significantly increases reaction rate by more than two orders of magnitude as compared to the use of no catalyst. This discrepancy may be caused by the effect of toluene, which remarkably increases the reaction rate compared to Et_2_O. From these observations, it seems that this asymmetric induction is not just a kinetic phenomenon in the reaction performed in toluene. Using quinine, a cinchona alkaloid, also provided the product (+)-**4** with 83% *ee* in 90% yield (entry 9).

**Table 2:** Optimization of reaction temperature and the amount of catalysts in toluene **^[^**^a**]**^. 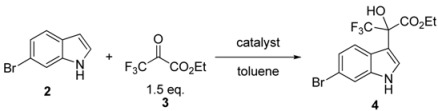

Entry	Catalyst (mol %)	Condition	Conversion ^[b]^		Yield (%)	*ee* (%)
			1 h	24 h		
1	CD (7.5)	−78 °C, 24 h	35	99	89	88
2	CD (7.5)	0 °C, 1 h	99	−	90	83
3	CD (7.5)	25 °C, 1 h	99	−	91	79
4	CD (7.5)	50 °C, 1 h	99	−	89	71
5	None	0 °C, 1 h	99	−	93	−
6	CD (1.0)	0 °C, 24 h	90	99	90	71
7	CD (2.5)	0 °C, 1 h	99	−	93	83
8	CD (15)	0 °C, 1 h	99	−	91	84
9	QN (7.5)	0 °C, 1 h	99	−	90	83

^[a]^ *Reaction conditions:* **2** (1.5 mmol), **3** (2.25 mmol) and catalyst in toluene (9 mL); ^[b]^ Conversion was determined on the basis of area % by HPLC (254 nm).

**Scheme 2:**
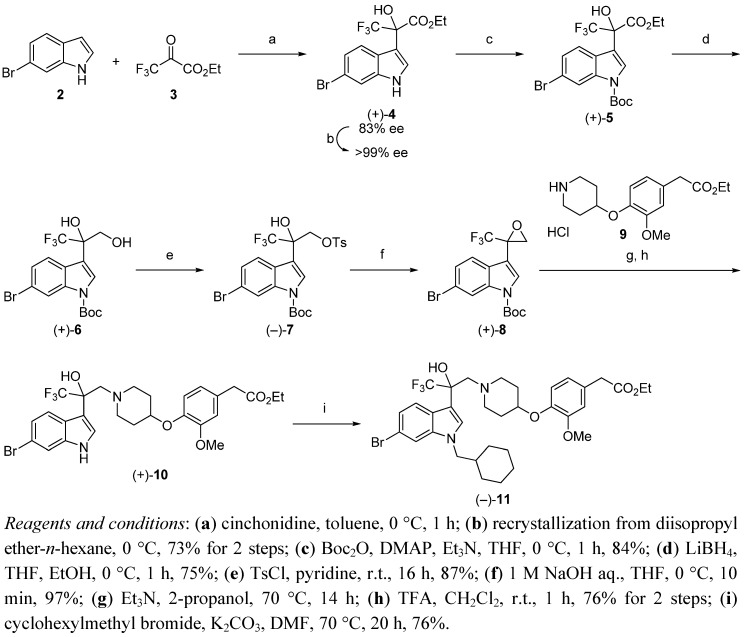
Synthesis of the intermediate (−)-**11**.

Based on the findings above, we synthesized the [^14^C]-labeled (−)-**1**. For a gram-scale synthesis of the enantiomerically pure **4**, toluene as solvent, 2.5 mol% CD, and 0 °C were selected as reaction conditions (Scheme 2). Under these conditions, the product (+)-**4** was obtained with 83% *ee* in 90% yield. Recrystallization of (+)-**4** from diisopropyl ether-*n*-hexane gave the racemic crystalline **4**, and the filtrate provided the enantiomerically pure (+)-**4** (>99% *ee*). The following reaction was performed according to the procedure previously reported for the 6-cyanoindole derivative [22]. *N*-Boc protection of the nitrogen atom of (+)-**4** with Boc_2_O gave (+)-**5**. Reduction of the ester (+)-**5** with LiBH_4_ gave the corresponding alcohol (+)-**6**. After that, compound (−)-**7** was prepared by tosylation of (+)-**6**. Epoxidation of (−)-**7** with aqueous 1 M NaOH in THF yielded (+)-**8**. Then, ring-opening reaction of **8** with the phenoxypiperidine **9** [22], followed by deprotection of the Boc group, provided (+)-**10**. Alkylation of (+)-**10** with cyclohexylmethyl bromide gave (−)-**11**.

Next, we considered establishing the cyano coupling with the 6-bromo group. For that, we selected CuCN and Zn(CN)_2_ as coupling reagents, because the corresponding [^14^C]-labeled reagents were available. As our attempt to perform the coupling reaction of CuCN to the 6-bromoindole moiety didn’t work well, we tried Negishi coupling on the ester (−)-**11** (Table 3). A simple attempt to introduce a cyano group by substitution reaction on the aryl bromide led only to decomposition of ester (−)-**11** (entries 1 and 2), thus we tried Negishi coupling using Pd(*t*-Bu_3_P)_2_ as catalyst (entries 3–5). Fortunately, running the reaction at 80 °C did not lead to the decomposition, but yielded 6-cyanoindole in 22% yield with recovery of (−)-**11** in 75% yield (entry 3). This result indicated that decomposition of the starting materials did not occur, allowing us to examine an increase in reaction temperature. Even at the highest temperature (110 °C) Negishi coupling conditions did not lead to product decomposition, but instead gave the desired (−)-**12** with recovery of (−)-**11** (entry 4). Among the temperatures tested, performing Negishi coupling at 150 °C afforded the product in 83% yield (entry 5). Applying the less reactive catalyst Pd(PPh_3_)_4_ to the reaction resulted in a lower yield (entry 6). Optimization of the reaction was achieved using [^14^C]-labeled Zn(CN)_2_ and afforded the corresponding [^14^C](−)-**12** in 78% yield (entry 7). As expected, hydrolysis of the obtained compound (−)-**12** resulted in the desirable enantiomer (−)-**1** with >99% *ee* in an overall yield of 9.3%. These results indicate that the established synthetic route caused no racemization.

**Table 3:** Optimization of cyano coupling **^[^**^a^**^]^**. 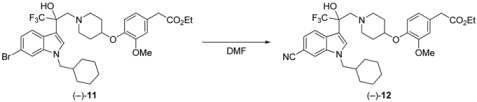

Entry	Reagent	Catalyst	Temprature (°C)	Time (h)	Yields (%)
1	2 eq. CuCN	non	100	3	−(decomposed)
2	2 eq. CuCN	non	170	2	−(decomposed)
3	2 eq. Zn(CN)_2_	0.2 eq Pd( *t*Bu_3_P)_2_	80	2	22 (75% recovered)
4	2 eq. Zn(CN)_2_	0.2 eq Pd( *t*Bu_3_P)_2_	110	2	78 (15% recovered)
5	2 eq. Zn(CN)_2_	0.2 eq Pd( *t*Bu_3_P)_2_	150	2	83
6	2 eq. Zn(CN)_2_	0.2 eq Pd(Ph_3_P)_4_	150	2	22
7	2 eq. [^14^C]Zn(CN)_2_	0.2 eq Pd( *t*Bu_3_P)_2_	150	2	78

^[a]^ *Reaction conditions*: (−)-**11** (43 μmol), Zn(CN)_2_ (86 μmol) and catalyst (8.6 μmol) in DMF (1.0 mL).

**Scheme 3:**
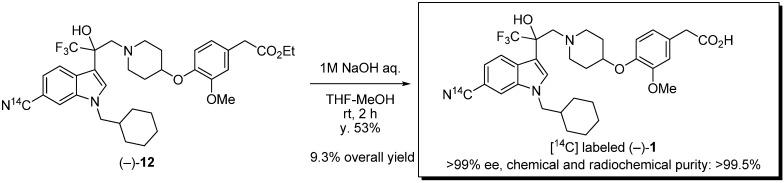
Synthesis of [^14^C] labeled (−)-**1**.

Finally, we carried out the hydrolysis of [^14^C] labeled **12**. As a result of the analysis, enantiometically pure and [^14^C]-labeled (−)-**1** was obtained in sufficient amount and radioactivity (Scheme 3).

## 3. Experimental

### 3.1. General

All reagents and solvents were used as obtained from commercial suppliers without further purification. Monitoring of reactions was carried out using Merck 60 F_254_ silica gel, glass-supported TLC plates, and visualization with UV light (254 nm). NMR spectra were recorded on a JEOL JNM-AL400 spectrometer (^1^H at 400 MHz and ^13^C at 100 MHz) at room temperature. Chemical shifts were given in *δ* values (ppm), and following abbreviations were used: s = singlet, d = doublet, t = triplet, q = quartet, dd = double doublet and m = multiplet. IR spectra were recorded on a PerkinElmer Spectrum One using the attenuated total reflection (ATR) technology. Optical rotations were recorded on a JASCO Polarimeter P-1020. Melting points were recorded on a Yanako MP-J3 melting point apparatus without correction. Low-resolution mass spectra were recorded on a Shimadzu LCMS-2010EV instrument under electron spray ionization (ESI) conditions. Elemental analyses were obtained on a CE Instruments EA1110. [^14^C] Zn(CN)_2_ (5.0 Ci, 115 mCi/mmol) was purchased from American Radiolabeled Chemicals, Inc. Radio-TLC was scanned on a raytest Rita Star. Quantitation of radioactivity was recorded on an AB Sciex 4000QTrap MS instrument. The following abbreviations are used for reagents and solvents: TFA (trifluoroacetic acid), Boc_2_O (di-*tert*-butyl dicarbonate), DMF (*N,N*-dimethylformamide), EtOAc (ethyl acetate), THF (tetrahydrofuran), IPE (diisopropyl ether).

*(+)-Ethyl 2-(6-bromo-1H-indol-3-yl)-3,3,3-trifluoro-2-hydroxypropanoate* (+)-**4**. To a mixture of 6-bromoindole **2** (294 mg, 1.5 mmol) and cinchonidine (11.0 mg, 37.5 μmol) in dry toluene (7 mL) was added ethyl trifluoropyruvate **3** (298 μL, 2.25 mmol) in dry toluene (2 mL) dropwise at 0 °C. The reaction mixture was stirred at 0 °C for 1 h. The mixture was quenched with MeOH (20 mL) and DMF (2 mL) and stirred at room temperature over 30 min. The mixture was concentrated and the residue was purified by silica gel chromatography (EtOAc/*n*-hexane = 1/2) to afford (+)-**4** (495 mg, 90%, 83% *ee*) as a pale brown oil; Anal. Calcd for C_13_H_11_BrF_3_NO_3_: C, 42.65; H, 3.03; N, 3.83. Found: C, 42.87; H, 3.04; N, 3.93.; ^1^H-NMR (DMSO-*d*_6_) *δ* 11.49 (s, 1H, NH), 7.64 (d, *J* = 8.7 Hz, 1H), 7.60 (d, *J* = 1.8 Hz, 1H), 7.51 (s, 1H, OH), 7.47 (d, *J* = 2.2 Hz, 1H), 7.17 (dd, *J* = 8.7, 1.8 Hz, 1H), 4.42–4.16 (m, 2H), 1.20 (t, *J* = 7.1 Hz, 3H); ^13^C-NMR (DMSO-*d*_6_) *δ* 167.8, 137.3, 126.1, 124.2 (q, ^1^*J*_C-F_ = 287 Hz), 124.0, 122.30, 122.26, 114.4, 114.3, 108.7, 76.6 (q, ^2^*J*_C-F_ = 29.8 Hz), 62.1, 13.9; IR (ATR) *ν* 3469, 3391, 1729, 1615, 1540, 1455, 1390, 1370, 1335, 1300, 1256, 1275, 1222, 1171, 1137, 1110, 1095, 1074, 1006 cm^−1^; MS (ESI) *m/z* 388 (M+Na)^+^; Reaction progress was monitored by UFLC at room temperature using a Shimadzu LC-20AD pump equipped with a Shimadzu SPD-M20A detector and a Phenomenex Kinetex C18 100A column (3 mm × 75 mm, 2.6 μm), eluted at 0.8 mL/min with a 20 min gradient (from 10% B to 90% B), where solvent A is water (0.05% TFA solution) and solvent B is acetonitrile (0.05% TFA solution). Enantiomeric excess was measured by HPLC at room temperature using a Shimadzu LC-10AT pump equipped with a Shimadzu SPD-10A UV detector and a Chiralpak AD-H column (4.6 mm × 250 mm, 5 μm), eluted with EtOH/*n*-hexane = 10/90, flow rate 1.0 mL/min, *λ* = 254 nm, retention times: (+)-isomer 14.2 min, (−)-isomer 23.3 min.

*10 g-Scale Synthesis of (+)-Ethyl 2-(6-bromo-1H-indol-3-yl)-3,3,3-trifluoro-2-hydroxypropanoate* (+)-**4**. To a mixture of 6-bromoindole **2** (10.0 g, 51.0 mmol) and cinchonidine (375 mg, 1.28 mmol) in dry toluene (80 mL) was added ethyl trifluoropyruvate **3** (10.4 g, 61.2 mmol) in dry toluene (20 mL) dropwise at 0 °C. The reaction mixture was stirred at 0 °C for 1 h. The mixture was concentrated and the residue was purified by silica gel chromatography (EtOAc/*n*-hexane = 1/2) to afford (+)-**4** (83% *ee*) as a pale brown oil. The residue was triturated with IPE (20 mL) and *n*-hexane (60 mL) at 0 °C. The mixture was filtered and the filtrate was concentrated to give (+)-**4** (13.6 g, 73%, 99% *ee*) as a pale brown oil; [α]_D_^25^ +12.4 (*c* 2.01, CHCl_3_).

*(+)-tert-Butyl 6-bromo-3-(3-ethoxy-1,1,1-trifluoro-2-hydroxy-3-oxopropan-2-yl)-1H-indole-1-carboxylate* (+)-**5**. To a mixture of (+)-**4** (9.3 g, 25.4 mmol, 99% *ee*), triethylamine (4.24 ml, 30.5 mmol) and 4-dimethylaminopyridine (310 mg, 2.54 mmol) in dry THF (38 mL) was added Boc_2_O (6.1 g, 27.9 mmol) in dry THF (13 mL) dropwise at 0 °C. The mixture was stirred at 0 °C for 1 h. The reaction mixture was neutralized with 5% KHSO_4_ solution. The mixture was extracted with EtOAc. The organic layer was washed with water and brine, dried over Na_2_SO_4_ and concentrated. The residue was purified by silica gel chromatography (EtOAc/*n*-hexane = 1/5) to afford (+)-**5** (9.9 g, 84%) as a pale brown solid; Anal. Calcd for C_18_H_19_BrF_3_NO_5_: C, 46.37; H, 4.11; N, 3.00. Found: 46.53; H, 4.04; N, 3.06; [α]_D_^25^ +15.2 (*c* 2.03, CHCl_3_); m.p. 54–55 °C; ^1^H-NMR (DMSO-*d*_6_) *δ* 8.27 (s, 1H), 7.97 (s, 1H, OH), 7.75 (s, 1H), 7.70 (d, *J* = 8.5 Hz, 1H), 7.46 (d, *J* = 8.5 Hz, 1H), 4.42–4.21 (m, *2*H), 1.61 (s, 9H), 1.20 (t, *J* = 7.1 Hz, 3H); ^13^C-NMR (DMSO-*d*_6_) *δ* 166.9, 148.2, 135.7, 126.3, 126.02, 125.99, 123.7 (q, ^1^*J*_C-F_ = 287 Hz), 123.1, 117.7, 117.5, 114.4, 85.2, 76.2 (q, ^2^*J*_C-F_ = 30.1 Hz), 62.7, 27.4, 13.8; IR (ATR) *ν* 3437, 1738, 1603, 1555, 1455, 1433, 1368, 1290, 1253, 1239, 1220, 1152, 1126, 1088, 1009 cm^−1^; MS (ESI) *m/z* 488 (M+Na)^+^.

*(+)-tert-Butyl 6-bromo-3-(1,1,1-trifluoro-2,3-dihydroxypropan-2-yl)-1H-indole-1-carboxylate* (+)-**6**. To a mixture of LiBH_4_ (1.68 g, 77.2 mmol) in dry THF (72 mL) and EtOH (9 mL) was added (+)-**5** (9.0 g, 19.3 mmol) in dry THF (18 mL) dropwise at 0 °C. The mixture was stirred at 0 °C for 1 h. The reaction mixture was quenched with 5% KHSO_4_ solution. The mixture was extracted with EtOAc. The organic layer was washed with water and brine, dried over Na_2_SO_4_ and concentrated. The residue was purified by silica gel chromatography (EtOAc/*n*-hexane = 1/2) to afford (+)-**6** (6.16 g, 75%) as a white solid; Anal. Calcd for C_16_H_17_BrF_3_NO_4_: C, 45.30; H, 4.04; N, 3.30. Found: 45.41; H, 3.98; N, 3.34; [α]_D_^25^ +12.1 (*c* 2.00, CHCl_3_); m.p. 120–121 °C; ^1^H-NMR (DMSO-*d*_6_) *δ* 8.24 (d, *J* = 1.7 Hz, 1H), 7.83 (d, *J* = 8.6 Hz, 1H), 7.74 (s, 1H), 7.42 (dd, *J* = 8.6, 1.7 Hz, 1H), 6.67 (s, 1H, OH), 5.28 (t, *J* = 5.8 Hz, 1H, OH), 4.00 (dd, *J* = 11.5, 5.8 Hz, 1H), 3.89 (dd, *J* = 11.5, 5.8 Hz, 1H), 1.62 (s, 9H); ^13^C-NMR (DMSO-*d*_6_) *δ* 148.5, 135.6, 127.6, 125.70, 125.66 (q, ^1^*J*_C-F_ = 288 Hz), 125.5, 124.0, 117.266, 117.258, 117.1, 84.9, 75.2 (q, ^2^*J*_C-F_ = 27.3 Hz), 63.4, 27.5; IR (ATR) *ν* 3410, 3294, 1707, 1606, 1557, 1465, 1454, 1430, 1383, 1371, 1331, 1310, 1289, 1271, 1252, 1189, 1156, 1135, 1109, 1083 cm^−1^; MS (ESI) *m/z* 446 (M+Na)^+^.

*(−)-tert-Butyl 6-bromo-3-(1,1,1-trifluoro-2-hydroxy-3-{[(4-methylphenyl)sulfonyl]oxy}propan-2-yl)-1H-indole-1-carboxylate* (−)-**7**. To a mixture of (+)-**6** (5.9 g, 13.9 mmol) in pyridine (47 mL) was added *p*-toluenesulfonyl chloride (13.3 g, 69.5 mmol) at room temperature. The mixture was stirred at room temperature for 16 h. The reaction mixture was concentrated, CHCl_3_ was added to the residue and washed with 2 M HCl (×2). The organic layer was washed with water (×2) and brine, dried over Na_2_SO_4_ and concentrated. The residue was purified by silica gel chromatography (EtOAc/*n*-hexane = 1/4) to afford (−)-**7** (6.99 g, 87%) as a white solid; Anal. Calcd for C_23_H_23_BrF_3_NO_6_S: C, 47.76; H, 4.01; N, 2.42. Found: 47.86; H, 3.93; N, 2.48; [α]_D_^25^ −10.8 (*c* 2.08, CHCl_3_); m.p. 118–119 °C; ^1^H-NMR (DMSO-*d*_6_) *δ* 8.20 (d, *J* = 1.7 Hz, 1H), 7.66 (s, 1H), 7.64–7.54 (m, 3H), 7.52 (s, 1H, OH), 7.33 (dd, *J* = 8.7, 1.7 Hz, 1H), 7.28 (d, *J* = 8.3 Hz, 2H), 4.57 (d, *J* = 10.6 Hz, 1H), 4.47 (d, *J* = 10.6 Hz, 1H), 2.38 (s, 3H), 1.63 (s, 9H); ^13^C-NMR (DMSO-*d*_6_) *δ* 148.2, 145.1, 135.4, 131.3, 129.8, 127.5, 126.4, 125.8, 125.7, 124.5 (q, ^1^*J*_C-F_ = 288 Hz), 123.1, 117.41, 117.36, 114.6, 85.2, 73.7 (q, ^2^*J*_C-F_ = 29.2 Hz), 69.3, 27.5, 21.1; IR (ATR) *ν* 3404, 1735, 1660, 1555, 1454, 1436, 1364, 1337, 1285, 1252, 1232, 1189, 1171, 1134, 1094, 1054, 1027 cm^−1^; MS (ESI) *m/z* 602 (M+Na)^+^.

*(+)-tert-Butyl 6-bromo-3-[2-(trifluoromethyl)oxiran-2-yl]-1H-indole-1-carboxylate* (+)-**8**. To a mixture of (−)-**7** (6.6 g, 11.4 mmol) in THF (57 mL) was added 1 M NaOH (11.4 mL) at 0 °C. The mixture was stirred at 0 °C for 10 min. The reaction mixture was neutralized with saturated NH_4_Cl solution. The mixture was extracted with EtOAc. The organic layer was washed with water and brine, dried over Na_2_SO_4_ and concentrated to afford (+)-**8** (4.51 g, 97%) as a white solid; Anal. Calcd for C_16_H_15_BrF_3_NO_3_: C, 47.31; H, 3.72; N, 3.45. Found: 47.42; H, 3.66; N, 3.49; [α]_D_^25^ +13.4 (*c* 2.08, CHCl_3_); m.p. 81–82 °C; ^1^H-NMR (CDCl_3_) *δ* 8.26 (d, *J* = 1.8 Hz, 1H), 7.99 (s, 1H), 7.61 (d, *J* = 8.5 Hz, 1H), 7.47 (dd, *J* = 8.5, 1.8 Hz, 1H), 3.57 (d, *J* = 4.5 Hz, 1H), 3.37–3.32 (m, 1H), 1.61 (s, 9H); ^13^C-NMR (CDCl_3_) *δ* 148.1, 135.1, 128.3, 127.0, 126.3, 123.4 (q, ^1^*J*_C-F_ = 278 Hz), 121.6, 117.9, 117.7, 110.5, 85.3, 53.0 (q, ^2^*J*_C-F_ = 38.1 Hz), 50.0, 27.4; IR (ATR) *ν* 1732, 1609, 1586, 1562, 1457, 1435, 1390, 1370, 1312, 1252, 1224, 1163, 1146, 1094, 1068, 1050 cm^−1^; MS (ESI) *m/z* 306 (M−Boc)^+^.

*(+)-Ethyl [4-({1-[2-(6-bromo-1H-indol-3-yl)-3,3,3-trifluoro-2-hydroxypropyl]piperidin-4-yl}oxy)-3-methoxyphenyl]acetate* (+)-**10**. To a mixture of (+)-**8** (4.3 g, 10.6 mmol) and Et_3_N (3.68 mL, 26.5 mmol) in 2-propanol (35 mL) was added **9** (3.84 g, 11.6 mmol) at room temperature. The mixture was stirred at 70 °C for 14 h. After cooling to room temperature, the reaction mixture was neutralized with 5% KHSO_4_ solution. The mixture was extracted with EtOAc. The organic layer was washed with water and brine. The organic layer was dried over Na_2_SO_4_ and concentrated. To a mixture of the residue in CH_2_Cl_2_ (17 mL) was added TFA (34 mL) at room temperature. The mixture was stirred at room temperature for 1 h. The reaction mixture was concentrated, EtOAc was added to the residue and the solution washed with saturated NaHCO_3_ solution and brine. The organic layer was dried over Na_2_SO_4_ and concentrated. The residue was purified by silica gel chromatography (EtOAc/*n*-hexane = 1/1) to afford (+)-**10** (4.81 g, 76%) as an amorphous solid; Anal. Calcd for C_27_H_30_BrF_3_N_2_O_5_: C, 54.10; H, 5.04; N, 4.67. Found: 54.26; H, 5.04; N, 4.73; [α]_D_^25^ +4.51 (*c* 2.09, CHCl_3_); m.p. 55–56 °C; ^1^H-NMR (400 MHz, DMSO-*d*_6_) *δ* 11.32 (d, *J* = 2.2 Hz, 1H, NH), 7.73 (d, *J* = 8.8 Hz, 1H), 7.56 (d, *J* = 1.7 Hz, 1H), 7.46 (d, *J* = 2.7 Hz, 1H), 7.12 (dd, *J* = 8.5, 1.7 Hz, 1H), 6.98–6.79 (m, *2*H), 6.70 (dd, *J* = 8.2, 1.8 Hz, 1H), 6.11 (s, 1H, OH), 4.20–4.09 (m, 1H), 4.05 (q, *J* = 7.1 Hz, 2H), 3.70 (s, 3H), 3.53 (s, 2H), 3.12 (d, *J* = 13.8 Hz, 1H), 3.00 (d, *J* = 13.8 Hz, 1H), 2.78–2.59 (m, 2H), 2.40–2.22 (m, 2H), 1.84–1.65 (m, 2H), 1.61–1.41 (m, 2H), 1.16 (t, *J* = 7.2 Hz, 3H); ^13^C-NMR (100 MHz, DMSO-*d*_6_) *δ* 171.3, 150.0, 145.1, 137.3, 127.6, 126.2 (q, ^1^*J*_C-F_ = 287 Hz), 125.4, 124.7, 122.9, 121.7, 121.3, 116.5, 114.1, 113.8, 112.6, 74.0 (q, ^2^*J*_C-F_ = 27.0 Hz), 73.3, 60.1, 60.0, 55.6, 51.8, 51.7, 39.9, 30.6, 22.1, 21.9, 14.1; IR (ATR) *ν* 3336, 1723, 1611, 1590, 1541, 1509, 1453, 1420, 1369, 1334, 1265, 1226, 1149, 1101, 1032 cm^−1^; MS (ESI) *m/z* 599 (M+H)^+^.

*(−)-Ethyl {4-[(1-{2-[6-bromo-1-(cyclohexylmethyl)-1H-indol-3-yl]-3,3,3-trifluoro-2-hydroxypropyl} piperidin-4-yl)oxy]-3-methoxyphenyl}acetate* (−)-**11**. To a mixture of (+)**-10** (4.12 g, 6.87 mmol), K_2_CO_3_ (5.70 g, 41.2 mmol) and KI (114 mg, 0.687 mmol) in DMF (69 mL) was added cyclohexylmethyl bromide (3.65 g, 20.6 mmol) at room temperature. The mixture was stirred at 70 °C for 20 h. After cooling to room temperature, the reaction mixture was diluted with water and extracted with EtOAc. The organic layer was washed with water and brine. The organic layer was dried over Na_2_SO_4_ and concentrated. The residue was purified by silica gel chromatography (EtOAc/n-hexane = 1/3) to afford (−)-**11** (3.61 g, 76%) as a pale brown oil; Anal. Calcd for C_34_H_42_BrF_3_N_2_O_5_·0.25H_2_O: C, 58.33; H, 6.12; N, 4.00. Found: 58.23; H, 6.01; N, 4.01; [α]_D_^25^ −4.65 (*c* 2.02, CHCl_3_); ^1^H-NMR (DMSO-*d*_6_) δ 7.81–7.67 (m, 2H), 7.43 (s, 1H), 7.13 (dd, *J* = 8.7, 1.8 Hz, 1H), 6.92–6.78 (m, 2H), 6.69 (dd, *J* = 8.2, 1.6 Hz, 1H), 6.13 (s, 1H, OH), 4.18–4.08 (m, 1H), 4.08–3.91 (m, 4H), 3.69 (s, 3H), 3.53 (s, 2H), 3.13 (d, *J* = 13.7 Hz, 1H), 2.94 (d, J = 13.7 Hz, 1H), 2.78–2.57 (m, 2H), 2.40–2.17 (m, 2H), 1.83–1.34 (m, 10H), 1.23–0.83 (m, 5H), 1.16 (t, *J* = 7.2 Hz, 3H); ^13^C-NMR (DMSO-d_6_) δ 171.2, 150.0, 145.1, 137.4, 129.5, 127.6, 126.1 (q, ^1^*J*_C-F_ = 287 Hz), 125.0, 123.1, 121.7, 121.3, 116.6, 114.1, 113.8, 112.9, 101.4, 74.2 (q, ^2^*J*_C-F_ = 27.3 Hz), 73.4, 60.1, 59.9, 55.5, 51.71, 51.65, 51.5, 39.9, 38.1, 30.7, 30.6, 29.93, 29.88, 25.9, 25.1, 14.0; IR (ATR) *ν* 3680, 1731, 1606, 1589, 1541, 1509, 1466, 1450, 1420, 1366, 1265, 1225, 1141, 1032 cm^−1^; MS (ESI) *m/z* 695 (M+H)^+^.

*(−)-Ethyl* *{4-[(1-{2-[6-[^14^C]cyano-1-(cyclohexylmethyl)-1H-indol-3-yl]-3,3,3-trifluoro-2-hydroxy-propyl}piperidin-4-yl)oxy]-3-methoxyphenyl}acetate* (−)-**12**. A mixture of (−)**-11** (30 mg, 43.1 μmol), [^14^C] Zn(CN)_2_ (5.1 mg, 42.0 μmol, 4.83 mCi, 115 mCi/mmol) and Zn(CN)_2_ (5.2 mg, 44.3 μmol) in dry DMF (1.0 mL) was stirred at room temperature for 30 min under argon. Pd(t-Bu_3_P)_2_ (4.4 mg, 8.61 μmol) was added to the mixture which was stirred at 150 °C for 2 h. After cooling to room temperature, the reaction mixture was diluted with saturated NaHCO_3_ solution and EtOAc. The mixture was filtered through Celite. The filtrate was extracted with EtOAc (×2). The organic layer was washed with brine, dried over Na_2_SO_4_ and concentrated. The residue was purified by silica gel chromatography (EtOAc/n-hexane = 1/3) to afford (−)-**12** (21.7 mg, 78%) as colorless oil. The data of unlabeled (−)-**12**; Anal. Calcd for C_35_H_42_BrF_3_N_3_O_5_: C, 65.51; H, 6.60; N, 6.55. Found: 65.43; H, 6.60; N, 6.47; [α]_D_^25^ −2.42 (*c* 2.06, CHCl_3_); ^1^H-NMR (DMSO-*d*_6_) *δ* 8.16 (s, 1H), 7.94 (d, *J* = 8.4 Hz, 1H), 7.72 (s, 1H), 7.33 (d, *J* = 8.4 Hz, 1H), 6.89–6.79 (m, 2H), 6.69 (dd, *J* = 8.4, 1.6 Hz, 1H), 6.29 (s, 1H, OH), 4.19–3.99 (m, 5H), 3.68 (s, 3H), 3.53 (s, 2H), 3.15 (d, *J* = 13.8 Hz, 1H), 2.94 (d, *J* = 13.8 Hz, 1H), 2.77–2.60 (m, 2H), 2.37–2.21 (m, 2H), 1.82–1.52 (m, 6H), 1.50–1.35 (m, 4H), 1.19–1.02 (m, 6H), 1.01–0.87 (m, 2H); ^13^C-NMR (DMSO-d_6_) δ 171.2, 150.0, 145.1, 135.4, 132.6, 129.1, 127.7, 126.1 (q, ^1^*J*_C-F_ = 287.6 Hz), 122.5, 121.3, 120.5, 116.6, 115.6, 113.8, 111.9, 102.5, 74.5 (q, ^2^*J*_C-F_ = 26.8 Hz), 73.4, 60.1, 59.9, 55.5, 51.8, 51.6, 39.8, 38.1, 30.7, 30.6, 29.9, 29.8, 25.9, 25.1, 14.; IR (ATR) *ν* 3680, 2220, 1731, 1615, 1589, 1541, 1509, 1471, 1451, 1420, 1388, 1367, 1332, 1264, 1226, 1139, 1097, 1033 cm^−1^; MS (ESI) *m/z* 642 (M+H)^+^.

*(−)-{4-[(1-{2-[6-[^14^C]cyano-1-(cyclohexylmethyl)-1H-indol-3-yl]-3,3,3-trifluoro-2-hydroxypropyl}-piperidin-4-yl)oxy]-3-methoxyphenyl}acetic acid* (−)-**1**. To a mixture of (−)**-12** (21.7 mg, 33.7 μmol) in THF (0.34 mL) and MeOH (0.34 mL) was added 1 M NaOH (0.34 mL) at room temperature. The mixture was stirred at room temperature for 2 h. The reaction mixture was neutralized with saturated NH_4_Cl solution. The mixture was extracted with EtOAc. The organic layer was washed with brine. The organic layer was dried over Na_2_SO_4_ and concentrated. The residue was purified by silica gel chromatography (MeOH/CHCl_3_ = 1/20) to afford (−)**-1** (11.0 mg, 53%, 57.0 mCi/mmol, >99% *ee*) as an amorphous solid. HPLC (retention time = 24.3 min) and radio-TLC (plate developed in 10% MeOH/CHCl_3_) showed the product to be more than 99.5% pure. Chemical and radiochemical purity was measured by HPLC at 40 °C using a Shimadzu LC-10AD pump equipped with a Shimadzu SPD-M20A UV detector (λ = 220 nm) and a PerkinElmer 150TR radioactivity detector with a Sumika Chemical Analysis Service Sumipax ODS A-212 column (6 mm × 150 mm, 5 μm), eluted at 1.0 mL/min with a 45 min gradient (from 20% B to 95% B), where solvent A is water (0.1% TFA solution) and solvent B is acetonitrile (0.1% TFA solution). Enantiomeric excess was measured by HPLC [Chiralcel OD-RH (4.6 mm × 150 mm, 5 μm), water (1% TFA solution)/acetonitrile (1% TFA solution) = 66/34, flow rate 1.0 mL/min, λ = 220 nm, retention times: (+)-isomer 30.8 min, (−)-isomer 33.3 min]. Co-injection of the radiolabeled product with an unlabeled standard of product (−)**-1** produced a single peak.

## 4. Conclusions

In this study we have optimized the asymmetric addition of 6-bromoindole (**2**) to ethyl trifluoropyruvate (**3**) and developed an efficient synthesis of enantiomerically pure key intermediate (+)-**4** (>99% *ee*) by using easily available, recyclable, and inexpensive cinchona alkaloids as catalysts. In addition, we discovered that Negishi coupling of zinc cyanide to the 6-bromoindole moiety proceeds smoothly without decomposition. Based on these results, we successfully synthesized [^14^C]-labeled GRM (−)-**1**. This study is the first example to apply organocatalyst-catalyzed reactions to asymmetric synthesis of an isotope labeled compound. This methodology is important in drug industry, because the numbers of enantiometically pure drug candidates is increasing and enantiomerically pure RI-labeled drug candidates are necessary for the drug development process. Our studies show that organocatalysts could be an important option for RI synthesis. 
